# Addressing AMR and planetary health in primary care: the potential of general practitioners as change agents

**DOI:** 10.3389/fpubh.2024.1383423

**Published:** 2024-07-31

**Authors:** Paula Tigges, Alexandra Greser, Ildikó Gágyor, Judith Kraft, Andy Maun, Guido Schmiemann, Eva-Maria Schwienhorst-Stich, Christoph Heintze, Angela Schuster

**Affiliations:** ^1^Charité – Universitätsmedizin Berlin, Institute of General Practice, Berlin, Germany; ^2^Department of General Practice, University Hospital Wuerzburg, Wuerzburg, Germany; ^3^Charité – Universitätsmedizin Berlin, Institute of Biometry and Clinical Epidemiology, Berlin, Germany; ^4^Institute of General Practice/Primary Care, Faculty of Medicine and Medical Center, University of Freiburg, Freiburg, Germany; ^5^Department of Health Service Research, Institute for Public Health and Nursing Research, University of Bremen, Bremen, Germany; ^6^Faculty of Medicine, Working Group Climate and Planetary Health, University of Würzburg, Wuerzburg, Germany

**Keywords:** planetary Health, antimicrobial resistance, primary care, change agents, general practice

## Abstract

**Introduction:**

Antimicrobial resistance is closely linked with the health and stability of environmental systems and therefore a challenge for the health of the planet. General Practitioners, owing to their trusted positions and close patient relationships, can play a crucial role in addressing antimicrobial resistance within the framework of Planetary Health. The goal of our study was to examine General Practitioners’ knowledge, attitude, and practice regarding the linkage of antimicrobial resistance with Planetary Health to understand their potential as agents of change in this domain.

**Materials and methods:**

We conducted 19 guided interviews with General Practitioners from four different German federal states (August–September 2022). Participants were selected from the intervention group of the RedAres randomized controlled trial, a study designed to optimize therapy and prescribing practices for uncomplicated urinary tract infections in general practice. Data were analyzed using Mayring’s structured qualitative content analysis and the typology approach by Kelle and Kluge.

**Results:**

General Practitioners generally demonstrated the ability to identify the interlinkages between antimicrobial resistance and Planetary Health. However, they exhibited varying levels of knowledge, problem awareness, and accountability for the associated challenges and partially outsourced the responsibility for Planetary Health. Some General Practitioners were capable of integrating Planetary Health arguments into patient counseling. They recognized rational prescribing practice, self-reflection on antimicrobial resistance and Planetary Health, interprofessional exchange, and raising awareness among patients as potential avenues for engagement in promoting Planetary Health.

**Discussion:**

As antimicrobial resistance is increasingly recognized as a Planetary Health challenge, empowering General Practitioners as change agents requires tailored measures based on their level of previous knowledge and their attitude toward Planetary Health. General Practitioners express a need for concrete advice on how to integrate antimicrobial resistance as a Planetary Health topic into their daily activities. Developing and evaluating adaptable training materials is essential. Additionally, the integration of Planetary Health outcomes into clinical guidelines could accelerate the adoption of this dimension in antibiotic prescribing practices within primary care settings.

## 1 Introduction

Antimicrobial resistance (AMR), a result of the excessive and improper use of antibiotics, is projected to cause 10 million deaths annually by 2050, unless effective countermeasures are implemented (1). AMR has an increasingly devastating impact on global healthcare and leads to shortages of the number of working antibiotics. Despite this looming crisis, antibiotic use continues to increase worldwide in human medicine and agricultural livestock management (2). Consequently, it is crucial to implement effective measures to combat AMR (3). In recent years, the interaction between AMR and the environment has been increasingly explored (4, 5). The World Health Organization (WHO) included AMR together with the climate crisis in its list of top 10 most serious threats to global health (6). The holistic concept of Planetary Health comprehensively encompasses the interrelationships among human health, animal health, and ecosystems, taking into account political, social, and economic influences. As outlined in the 2015 Rockefeller Foundation report, the goal of Planetary Health is to acknowledge the interdependencies between human health and the intactness of natural systems. It aims to promote health care and prevention in a sustainable manner for both people and the environment (7).

**Figure 1:**
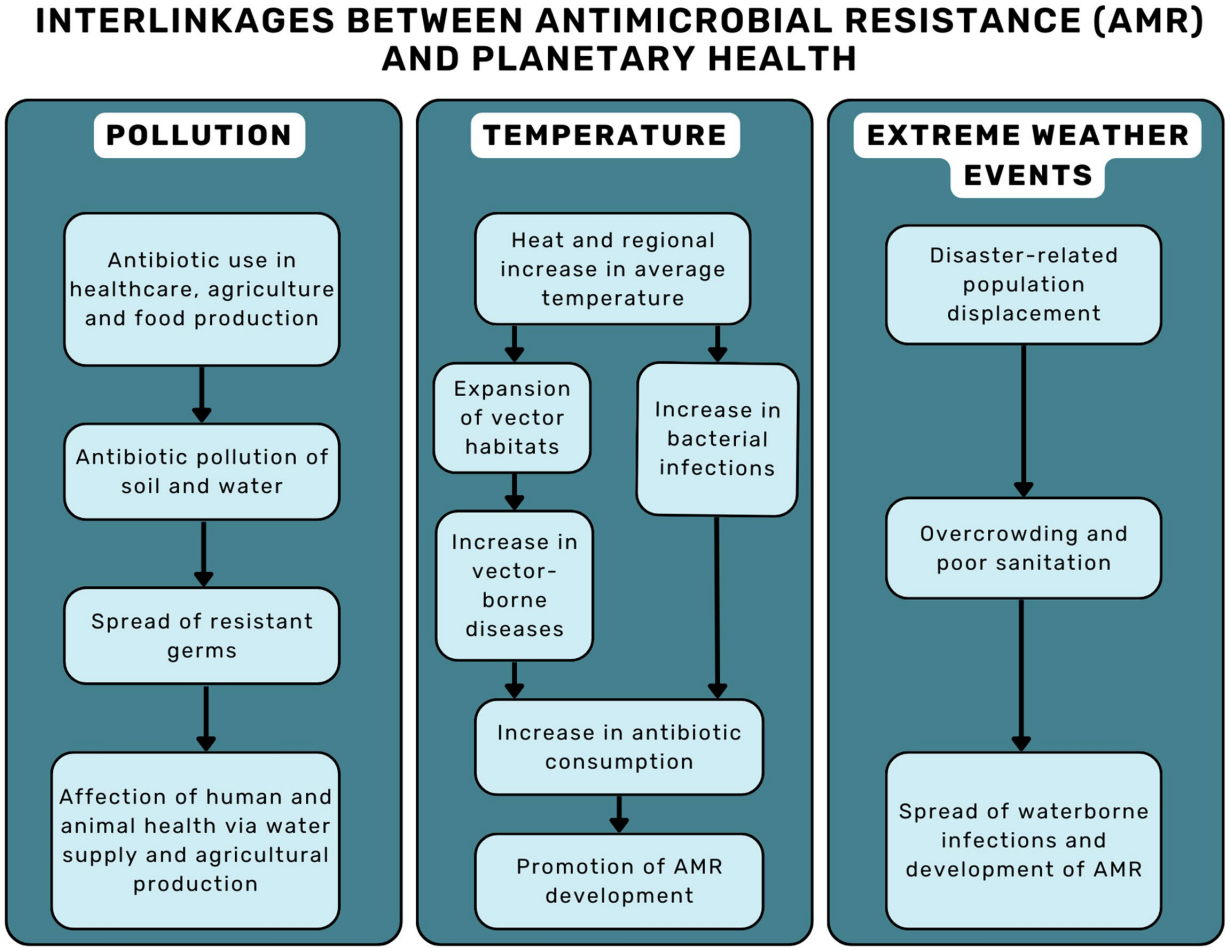
Interlinkages between AMR and planetary health.

Primary discussions on the interlinkages between AMR and Planetary Health have focused on three main aspects: (I) the pollution of soils and waters by antibiotics (4, 8, 9), (II) the effects of heat and the regional increase in average temperature (10–12), and (III) the consequences of extreme weather events (10, 12) (see Figure 1).

In this complex landscape, social tipping dynamics offer an interesting perspective on how to address these interlinked and urgent challenges. Similar to climate tipping points, which trigger cascades of environmental changes once a threshold is crossed (13); domino effects can also be initiated into a positive direction. Social tipping points (STPs) are small societal changes that trigger broader, large-scale shifts within a social-ecological system. This process is reinforced by positive feedback mechanisms, resulting in a fundamental qualitative change in the social-ecological system (14). The transformation of norms, values, and the education system can be viewed as social tipping interventions (STIs) that lead to the onset of sustainable change (15). When applied to the health care sector, presenting the climate crisis as a health issue is believed to potentially strengthen support for climate protection policies (16, 17).

General Practitioners (GPs) play a crucial role as catalysts for change in social tipping interventions, serving as key figures capable of initiating and implementing these interventions. GPs working in primary care have a close relationship to their patients and directly witness the health impacts of environmental change (18, 19). Additionally, they hold the responsibility for preventive healthcare (19). Given their position, they can function as intermediaries between various stakeholders. Within their practice, GPs acquire insights into a community’s needs, challenges, and the social and environmental factors influencing health and vulnerabilities (19). Drawing on their experience and the trust placed in their profession (20–22), GPs can actively promote Planetary Health in their practice and support sustainable behaviors and mitigation strategies (19). At the community level, GPs can foster or initiate collaborative efforts, such as advocating in local community activity groups (19), thereby enhancing the community’s health resilience (23). GPs can also act as advocates for Planetary Health by embedding their knowledge into regional, national or global policies (23). This strategic involvement allows them to leverage their social capital (24), effectively bridging the gap between individual health and environmental stewardship.

Currently, there is limited research on the extent to which GPs in Germany are aware of the links between the development of AMR and climate change. However, it is crucial to investigate the perspectives and potential for change among GPs to address the pressing challenges of AMR and Planetary Health (23). In this context, we aim to fill this research gap by examining the knowledge, attitudes, practical skills, and ideas held by GPs concerning the interrelations between AMR and Planetary Health. Additionally, we seek to explore their potential for acting as agents of change in addressing these critical issues.

## 2 Materials and methods

For quality assurance, we followed the Consolidated Criteria for Reporting Qualitative Research (COREQ) for reporting our methods (25).

### 2.1 Study design

The study was embedded in the process evaluation of the RedAres study, a randomized controlled trial (RCT) designed to optimize therapy and prescribing for uncomplicated urinary tract infections (UTIs) within GP practices across four regions in Germany (Berlin-Brandenburg, Thuringia, Baden-Wurttemberg, and Bavaria) (26). A total of 128 GP practices, comprising 64 interventions and 64 controls, were included in the data collection. The study included four interventions: provision of guideline information materials, the display of national and regional UTI resistance data from the Robert Koch Institute, individual prescription feedback, and benchmarking against the average prescriptions within the cohort. The impact of the interventions of antibiotic prescribing have been previously published (26).

The process evaluation of the RedAres study involved cross-sectional questionnaires and 19 qualitative interviews with GPs from the intervention group (27, 28). Within the framework of the qualitative interviews of the process evaluation, a subset of interviews specifically focused on AMR and Planetary Health, involving the same cohort of primary care physicians.

### 2.2 Study setting and population

The sample was based on the voluntary participation of GPs from the intervention group of the RedAres study. Potential participants were approached during the final visit by a member of the study team.

General Practitioners who expressed interest provided consent for the interviewer to contacted them through email or a phone call. To maximize variability in underrepresented regions and genders, we actively recruited participants from these groups after the initial interviews. A compensation incentive of 105€, irrespective of interview length, was offered for participation.

We conducted and audio-recorded all interviews online via Microsoft Teams video call in accordance with data safety regulations of our institution. The interviews were conducted in German, only the interviewer and the interviewee being present. All interviews were conducted between August 17 2022 and September 30 2022. Data saturation was achieved after 19 interviews and no interviews were repeated.

An interview guide was developed deductively based on the Knowledge, Attitude, Practice (KAP) (29, 30) framework. Utilizing this framework enabled the investigation of GPs’ perspectives on AMR in connection with Planetary Health by analyzing their pre-existing knowledge, attitudes, and practical skills. The interview guide underwent iterative adaptation after piloting with four primary care physicians and researchers at the Institute of General Medicine of the Charité and will be made available in full on request.

Throughout the interviews, field notes were taken to assist the interviewer’s memory and facilitate data analysis. Out of the conducted interviews, five were transcribed by the interviewer, while an additional 14 were transcribed by a commercial transcription agency. Data safety regulations were always respected. Transcripts were not returned to participants for member checking.

### 2.3 Data collection and analysis

The interviews were conducted and coded by PT (female, third-year medical student, doctoral candidate and student assistant in the RedAres project). Out of the 19 interviews, 10 were counter coded by AS (female, general practitioner, and public health researcher) and Zoe Friedmann (another female medical student). The study was supervised by AS and CH, both general practitioners and public health researchers. All researchers possessed prior experience in qualitative research, either through previous research or participation in the qualitative research network at the Institute of General Practice, Charité—Universitätsmedizin Berlin.

No personal or other relationship existed between the interviewer and the interviewee other than email contact to arrange the appointment. The interviewer identified herself as a medical student, doctoral candidate, and student assistant affiliated with the RedAres project. While conducting the interviews, she served as a student assistant in the RedAres project. She had a positive attitude toward the RCT’s aims and evidence-based medicine (EBM). In general, she had personal interest in Planetary Health and political awareness regarding the health consequences of climate change and social inequality in the field of medicine.

The data were analyzed based on Mayring’s structuring qualitative content analysis (31), using a mixed inductive-deductive approach. The deductive categories of the coding tree were formed using the KAP structure. During the analysis, additional subcategories were inductively created.

The codebook provides an overview of the inductive and deductive categories (Supplementary material 1). Data management and analysis were performed using MAXQDA 2022.

Utilizing Kelle and Kluge’s stage model of empirical development of typologies (32), we employed an inductive approach through interviews and field notes to identify four distinct types of GPs sharing similar traits. In the initial stage, we formulated relevant comparative dimensions, evaluating interviewees across three primary dimensions: knowledge, attitude, and practice. We assessed their amount of prior knowledge and associative competences. For attitude, we assessed problem awareness for AMR, accountability for Planetary Health, and their mindset toward problem-solving. Regarding practice, we assessed and compared experiences in integrating AMR and Planetary Health into daily work. Moving to the second stage, we analyzed and empirically clustered the cases based on identified patterns. Extracting and summarizing key points allowed us to categorize participant statements based on common characteristics within the KAP categories. In the third stage, the content-related connections and differentiations were analyzed, and the various GP types were developed. These types were then consolidated through a comprehensive re-evaluation of the characteristic statements.

### 2.4 Ethics

Ethics approval was obtained at the Ethics Committee of the Medical Faculty, University of Wuerzburg in November 2019, under the number 20191106 01. Data safety complies both with European data protection laws and Charité regulations. All respondents signed an informed consent form prior to the interview. In January 2020, the RedAres study was registered at the Trial registration site DRKS under the trial registration number DRKS00020389.

## 3 Results

Of the 64 RedAres intervention practices, 32 provided consent for interviews. Thirteen practices initially interested withdrew their participation; three cited lack of time, while the remaining 10 could not be contacted via phone or email.

Nineteen GPs were interviewed, with the interviews lasting between 46 and 87 min, averaging 62 min. The interviewed GPs comprised eight females and 11 males, originating from four different regions in Germany, with an average age of 51 years (range 34–74 years). The overview of the included GPs and their sociodemographic characteristics is described in detail in the publication of the RedAres process evaluation (27).

### 3.1 Knowledge: knowledge base and associations on planetary health interrelationships

The majority of GPs were not familiar with the term “Planetary Health,” but they conceptually connected it to their pre-existing knowledge of “One Health” (the linkage between human and animal health) and “Global Health.” Frequently, the term Planetary Health was linked to the rapid transmission pathways in our progressively globalized world. Specifically, they referred to the global spread of vector-borne infectious diseases from other countries, migration, and especially, the rapid proliferation of resistant germs.

*“Or when I see […] data […] from other countries, […] obviously […] not much attention is paid to it, then I think […] we live in a globalized world, no matter what pops up somewhere, it potentially spreads. And that has been seen quite blatantly with Corona, but that applies to all kinds of pandemics” (P4.1, male, 65 years).*

Numerous GPs established a strong connection between AMR and Planetary Health, emphasizing the linking factor of animal breeding. Additionally, several GPs raised concerns about water pollution attributed to the prophylactic use of antibiotics in animal farming and the manufacturing of antibiotics.

*“So, the first term is animal agriculture. […] That’s what I immediately think of when I think of antibiotic and Planetary Health. The next thing is antibiotic production, like India and polluted lakes and other things. And just there, just in the lakes alone, cultivation of resistant bacteria” (P3.7, male, 56 years).*

### 3.2 Attitude: problem awareness and positioning on accountability

General Practitioners expressed concerns about the interplay of multiple interconnected and escalating problems associated with the emergence of AMR. Frequently, they conveyed feelings of helplessness and perplexity, particularly in relation to the climate crisis. Many GPs expressed worry about the potential scarcity of antibiotic treatment options in the future and the inability to find solutions.

*“I see the rivers with the antibiotic fish down there. […] [T]he environment, which we are also affecting quite badly and simply harming ourselves in the long run. […]There are already fears, how will it be done in the future? Where is the research heading? Do we still have that many options? Do we still have enough room to come up with new therapeutic approaches?” (P2.4, female, 45 years).*

In fewer cases, AMR was not seen as a major threat. Some GPs shared their impression that especially in primary care; AMR is already being adequately addressed.

*“I think the family doctors are already aware of this problem [AMR development], that antibiotic prescriptions should be given less frequently and that we should first wait and see” (P2.2, male, 40 years).*

Some physicians tended to attribute the primary responsibility for AMR to other medical professionals, while regarding their own prescribing practice as correct.

*“It’s not so much the primary care physicians, it’s more the specialists who keep pushing it” (P3.5, female, 50 years).*

Furthermore, many GPs believed that the development of antibiotic resistance was linked to the perceived less restrictive use of antibiotics in countries of the Global South.

*“Major antibiotic resistance comes […] from other countries […] where antibiotics are prescribed much more easily, and where you do not have the same access to a doctor as here, and where you can actually buy antibiotics in the supermarket” (P4.3, male, 34 years).*

Several GPs expressed a strong sense of responsibility and acknowledged that their work influenced Planetary Health. They articulated a general responsibility for community health, with some specifically acknowledging a heightened sense of accountability for preventing AMR in primary care. This sentiment stemmed from the perception that GPs are responsible for prescribing the majority of antibiotics.

*“I try […] not only to have the individual in front of me in the focus, but see my responsibility as a general practitioner in the fact that I am also responsible for the general public and must […] always weigh it up. And that refers to any medication prescription, to any diagnostic measure” (P4.5, female, 41 years).*

Other GPs took the position that creating awareness of these larger health contexts is not their responsibility. Additionally, some questioned the significance of the impact when educating patients.

*“I think […] these very overarching topics have […] no place in the family doctor’s practice. […] Of course we also have an educational function. […]. But not to the extent that it is really relevant now” (P1.1, female, 54 years).*

Several GPs placed the responsibility for AMR more on the side of veterinary medicine and agriculture rather than human medicine. Several GPs, either predominantly or exclusively, identified the issue as arising from the use of antibiotics in agriculture and animal breeding.

*“So, I think that’s a much bigger factor […], animal husbandry, agribusiness, than medicine. I think my behavior is very small light there” (P3.1, male, 45 years).*

### 3.3 Practice: options and barriers for action

#### 3.3.1 Options for action inside the GP practice: self-reflection, rational prescribing and creating awareness

For many GPs, being aware of the connections between their practice and Planetary Health was very important. They viewed this awareness as a fundamental step in incorporating Planetary Health into their work. One given example was that recognizing the presence of medication residues in the environment would foster restrained use of antibiotics.

*“Yes, […] my actions are always determined by larger questions as well […]. For example, with antibiotics, […] I think about the fact that everything we use will eventually be floating around in our drinking water […], it will show up again in the animals. Also in the plants. […] That’s why I try […] to handle my work in such a way that only the most necessary things end up there” (P3.8, male, 52 years).*

Several interviewees stated that they actively stay informed about new clinical recommendations. They described utilizing various media sources, such as trade press, training sessions, and professional exchanges, to stay current and ensure their practice remains up to date.

*“[T]here are also constantly new developments that you have to keep up with somehow. [I: How do you stay up to date?] Through discussions with experts, through publications. […] I look at the literature again and again to see what’s new. You hear about it [AMR] at training courses. And attention is also repeatedly drawn to it in [the] specialist press […]” (P2.3, male, 63 years).*

A small number of interviewees expressed strong resistance to the concept of Planetary Health framing. They contended that an integration of Planetary Health considerations would not align with providing effective treatment of an individual patient. According to their perspective, incorporating a collective dimension into a treatment rationale could compromise the individual patient’s benefit.

*“[I: To what extent do you also think about these larger contexts of Planetary Health when prescribing antibiotics?] Certainly not, no. […] When I prescribe an antibiotic, I think about the patient sitting in front of me. […] I want him to get better. And not that he has some complication, that I have to send him to the clinic 3 days later and that he has completely different problems” (P1.3, female, 62 years).*

Numerous GPs viewed the judicious and proper use of antibiotics as a viable avenue for contributing to Planetary Health. They emphasized the significant role that GPs play in this regard, emphasizing that, as primary care providers, they are responsible for initiating and guiding the first line therapy.

*“[GPs play] an important role because we are usually the first contact person for the patients. We already decide on the first-line therapy […]. And if you already set the right course, that is of course a crucial point” (P1.1, female, 54 years).*

In this context, one GP emphasized the importance of directing attention to areas where changes in medical practice can have the most substantial impact on a global scale. The GP highlighted that a significant reduction in prescription medications can lead to a substantial decrease in CO_2_ emissions.

*“And also […] raise awareness again, how are the connections and […] where do I have the greatest impact when I try to change something, For example, medication, that there […] I could achieve the greatest impact […] for CO_2_ savings, because that is one of the largest CO_2_ emitters in everyday practice” (P4.5, female, 41 years).*

Some GPs also expressed the desire for support through guidelines advocating for more restrained antibiotic prescribing practices, emphasizing the importance of such measures in preserving Planetary Health.

*“I believe that a lot can still be done in many practices if antibiotics are handled more carefully and if we take a closer look at the recommendations. […] It would perhaps also make sense if we had a certain legal backup, and the guidelines are incredibly important here” (P4.1, male, 65 years).*

Other GPs underscored their crucial role as trusted communicators with their patients regarding all health-related issues, setting themselves apart from specialists in this aspect. They emphasized the believe that GPs have the ability to exert both short-term and long-term influence on their patients.

*“If we argue this well, then my words are still more important than those of the specialist colleague sometimes. Because they […] accept our advice more. We have a completely different position or trust relationship with the patients because we have known them for years and many then also consult us a second time” (P3.5, female, 50 years).*

The interviewed GPs expressed confidence in their ability to discuss the topic of AMR with their patients and effectively communicate its significance, especially when the subject is also covered by other sources of information.

*“If they read in the media 3 days later that in pig breeding or something like that a lot of antibiotics are simply given […] in the feed, […] or for the chicken, then they can perhaps also reflect on it better if the doctor has perhaps said this at some point beforehand” (P3.6, male, 38 years).*

Numerous GPs stated that the socioeconomic background of the patient and their (presumed) interest in the topic influence whether and how Planetary Health is discussed during the consultation.

*“It always depends […] on what kind of patient I see. How do I assess him? How far does he think? How does he think? Can I talk to him about the big picture? Is it better […] to talk about the small frame?” (P4.1, male, 65 years).*

Physicians noted that discussions about AMR arise particularly when the justification for prescribing antibiotics is uncertain. Some GPs find a promising strategy in highlighting the concept of co-benefits—connecting both individual and planetary advantages. They reported linking the personal benefits of cautious antibiotic prescribing with broader advantages for Planetary Health. This approach aims to enhance patient understanding and support for more restrained prescription practices.

*“If it’s a consideration that I say: ‘Well, you could give an antibiotic now, but you could perhaps also observe it a bit […]’, then I also discuss […] this larger level […]: ‘If you take antibiotics, then you are not only doing good for yourself, but you are also doing something bad for yourself […]. In terms of resistance. And you are also doing something bad for your environment, because of course you are also promoting the development of resistance […]’. And that is often a good balance, […] okay, […] you just have to […] maybe endure it a little longer, and in return I also gain something. […] I also have less risk of resistance” (P4.1, male, 65 years).*

#### 3.3.2 Options for action outside the GP practice: exchange in the GP and interprofessional context

Several GPs reported engaging in discussions about AMR and Planetary Health with their colleagues, empowering them to address these issues during patient consultations. They frequently highlighted that advanced trainings or quality circles serve as valuable opportunities to enhance awareness within the medical community.

*“Then a bit on the larger scale, by trying to talk to other colleagues about it, to take them to further training, […] to simply raise awareness of this problem a bit more, to bring it into the quality circle” (P4.5, female, 41 years).*

Others expressed their appreciation for self-organized networks of GPs, facilitating knowledge exchange and collaborative efforts to enhance the integration of Planetary Health into their practice. They emphasized that establishing such structures requires leadership and change agency within the community of GPs.

*“So, there would be a kind of self-help group for physicians who are interested in what can be done differently in practice. Because you just do not think of a lot of things. You have ideas, but maybe you do not dare to implement them […]. But with someone who is passionate about it and takes over a bit of the leadership, […] who perhaps already has a lot of experience and would like to share it. […] [T]here must already be someone who then deals with it intensively” (P3.4, female, 52 years).*

Some physicians expressed a desire to enhance interprofessional collaboration to address problems like AMR and promote Planetary Health more successfully. Pharmacists were frequently cited in this context, with GPs noting that improved cooperation with pharmacies could lead to reduced antibiotic and resource usage.

*“The GP practices […] [should] cooperate well with their local pharmacies […]. That one also checks with the pharmacies from time to time: ‘How about that? Does that work for the patient?’ Or: ‘Is this available in a different package size […]?’” (P3.4, female, 52 years).*

#### 3.3.3 Barriers to action: lack of education, economized health care system, and patients’ expectations

Some GPs criticized the training of physicians in antibiotic prescribing, noting that the training during residency in the hospital is not tailored to outpatient care. This mismatch may contribute to the perception that too many and overly broad antibiotics are prescribed in the outpatient sector, possibly indicating inadequate physician education. Additionally, some GPs shared the impression that physicians with less experience might prescribe less restrictively due to concerns about insuring adequate treatment for infections.

*“We are a training practice. We have young doctors who come from the hospital. There they learn how to handle antibiotics […]. And there is unfortunately only very poorly accessible information on the correct antibiotic prescription of the outpatient medicine outside of the hospital” (P3.1, male, 45 years).*

Some GPs voiced concerns about the perceived dominance of pharmaceutical companies in the training provided for physicians. They criticized the profit-oriented nature of these training programs, perceiving them as limited to lucrative therapeutics. Some GPs concluded that there is an imbalance, with limited continuing education on antibiotic prescribing as it is not viewed as financially profitable.

*“The problem […] is also that a lot of our training capacity is in the hands of pharmaceutical companies […]. And antibiotics are not the favorites of the pharmaceutical industry. […] You will […] maybe get one training [a year] on antibiotic treatment, if at all […]. So, […] advanced antibiotic training, who’s going to fund that […]?” (P2.3, male, 63 years).*

The majority of GPs described experiencing significant pressure due to time constraints during consultations and high patient throughput. Some GPs linked this pressure to less restrictive antibiotic prescribing practices, explaining that prescribing medication instead of discussing its indication and the Planetary Health effects, such as AMR, can expedite the consultation and save time.

*“[T]here is a high throughput, you also have to get people out again quickly, and then reaching for the antibiotic is often the faster solution, because what we would have to do, advice on phytopharmaceuticals, or symptomatic therapies, that costs time […], and that is the problem […], the talking medicine is not well paid” (P3.5, female, 50 years).*

General Practitioners found it challenging to integrate this additional topic into their practice due to time constraints and patient demands.

*“[S]ince family medicine […] is under great [time] pressure, I do not see the possibility of adding other topics to the list […]. I’d rather accept a new patient than to talk about Planetary Health for a longer time with a patient” (P2.2, male, 40 years).*

Several GPs observed that a significant number of patients arrive at consultations with pre-existing expectations of being prescribed antibiotics. It was assumed that these expectations often stem from misinformation about the efficacy of antibiotics and previous experiences with complication-free antibiotic treatments. Some GPs acknowledged that these strong patient expectations can impact their decision to prescribe antibiotics.

*“Of course, there are those […] who insist on it. It also has a placebo effect when they take their antibiotic, which helps immediately. So, you are a bit ambivalent, do you do it to silence them, or do you fight it out? Of course, this requires more counseling. With some people, however, you are not successful. Well, there are always those who are only happy when they get the stuff” (P3.5, female, 50 years).*

### 3.4 GPs typologies in navigating the interplay of AMR and planetary health

We categorized GPs into four types based on their knowledge, attitude, and behavior concerning AMR and Planetary Health in primary care. Table 1 outlines their specific features and needs.

**Table 1:** Types of GPs and their specific needs.

Type	Knowledge	Attitude	Practice	Needs
Type 1: rejecting	Lacking	Medium level	Lacking	
		Weak problem awareness. Partly trivializing, paternalistic. Rejection of accountability of GPs.	No reference to own prescription practice. No integration of Planetary Health arguments.	Wish for problem solution by other health care institutions.
	*“I do not have much of an idea about the term […]. But simply that you look at the whole thing in the big picture” (P1.1, female, 54 years).*	*“I’m too modest for that. I do not think about the planetary things. […] But nobody would want [global influence] either” (P1.2, male, 56 years).*	*“[I: To what extent do you bring Planetary Health into your consultation?] Rarely. […] I’m not there to give lectures or go through the lifestyle in detail” (P1.1, female, 54 years)*.	*“I think that these […] topics may unfortunately not have a proper place in a GP practice. Perhaps it’s more up to the school, the university, the training center to introduce young people to these topics” (P1.1, female, 54 years).*
Type 2: resigned	Medium level	Medium level	Low level	
		Moderate or strong problem awareness Threatened, pessimistic, frustrated, skeptical Attribution of accountability to other stakeholders of the health system	In favor of evidence-based prescribing Limited integration of Planetary Health arguments to justify rational antibiotic use in consultation Emphasis on time pressure and the concern of patronizing patients	Training offers from scientific, economically independent institutions
	*“The first thing that comes to mind is […] hygienic conditions, drinking water supply and food supply” (P2.3, male, 63 years).*	*“I feel like these societal decisions […] are a major cause of the problem and that I cannot influence them […]. Education is certainly important, but […] I’ve become a bit pessimistic about that” (P2.1, female, 41 years).*	*“I am skeptical. […] If there is a specific case, you speak out against antibiotics and then you must argue that, […], then I do see the possibility. But I do not see an explicit Planetary Health consultation as possible in the current time situation” (P2.2, male, 40 years).*	*“Independent training from interested parties or from institutes […]. Medical self-administration […], the universities […] [or] state bodies such as health authorities [could offer something like this]. Independent institutes, training institutes could also be set up to offer this” (P2.3, male, 63 years).*
Type 3: unconscious and open-minded	Medium level	High level	Medium level	
		Strong problem awareness. Open-minded and interested. Partial acknowledgement of responsibility of GPs for own patient clientele. Partial attribution of accountability to other stakeholders in the health system.	In favor of evidence-based prescribing. Occasion-related, patient-dependent integration of Planetary Health arguments to justify rational antibiotic use in consultation. Partial unconscious introduction of the topic without using the Planetary Health framing. Emphasis on time pressure and the concern of patronizing patients.	Training offers regarding the interlinkages between AMR and Planetary Health. Concrete tips for action regarding education.
	*“These pharmaceuticals [antibiotics] also have an impact on nature. […] There are connections between the environment and […] medication and medical practice” (P3.2, male, 74 years)*.	*“The GPs, […] [and] the veterinarians. […] Those are the two that are important. […] The other point is to improve the healthcare system. And then we would probably soon have the resistant germs under control” (P3.7, male, 56 years)*.	*“First, you look at […] the patient when you prescribe the antibiotic. […]But I think if you reflect on it critically, the other things [the inclusion of Planetary Health] come naturally” (P3.4, female, 52 years)*.	*“GPs, like me, need to know exactly: What are the areas where we should and can educate? Where are sensible intersections where the two things can perhaps be combined? The individual health advice, but also the link to Planetary Health” (P3.6, male, 38 years)*.
Type 4: motivated and resilient	High level	High level	High level	
		Strong problem awareness. Concerned, but resilient and motivated. Acknowledgement of the necessity of systemic changes, linked with acknowledgement of responsibility for own patient clientele.	In favor of evidence-based prescribing. Regular integration of Planetary Health arguments to justify rational antibiotic use in consultation, Co-Benefit arguments are used. Partly voluntary engagement for Planetary Health outside of the work context.	Compulsory integration of AMR and Planetary Health into education systems. Changing framework conditions to take planetary health more seriously.
	*“How to maintain the health of all living creatures on this earth and the health of our climate […]. By ensuring that humans, who exert the greatest influence of all living beings, think about the interactions between the individual living beings” (P4.2, female, 41 years).*	*“It’s our job to talk to the patients about it [Planetary Health contexts], even if they do not necessarily come with that concern” (P4.1, male, 65 years).*	*“Primarily […] to make the patient understand why I am so critical about prescribing antibiotics […] Then you must weigh things up with the patient […] and, […] take them along with you in a shared decision-making process […]. On the other hand, educating colleagues […] in quality circles or […] mixed GP get-togethers, where I’ve also given a presentation on this topic” (P4.2, female, 41 years).*	*“If we set framework conditions that show that we as a society are taking this issue seriously […], then we as doctors […] have a great influence in making this a recurring theme” (P4.1, male, 65 years).*

#### 3.4.1 Type 1: The rejecting type

General Practitioners classified as the “rejecting type” exhibited a lack of knowledge about AMR and Planetary Health, including their interrelations. Three GPs were included in this type.

They also demonstrated a relatively weak problem awareness regarding these topics. Additionally, a portion of “type 1 GPs” exhibited dismissive and paternalistic attitudes toward the role of primary healthcare in the development of AMR and associated Planetary Health concerns. This subset refused to acknowledge their responsibility in addressing these issues.

Moreover, GPs of the “rejecting type” refrained from incorporating Planetary Health arguments into consultations. This was either due to a lack of effective strategies to raise patients’ awareness or an active rejection of this responsibility.

#### 3.4.2 Type 2: The resigned type

The “resigned type” included four GPs with a medium level of prior knowledge about Planetary Health. Even if unfamiliar with the term, they demonstrated the ability to correctly define and deduct it, recognizing the links between AMR and Planetary Health.

Within the “Type 2 GPs,” there was a moderate level of problem awareness, with many acknowledging the profound implications of AMR for Planetary Health, considering it as a significant threat. Most in this group advocated for a systemic approach to address AMR, often shifting the responsibility to other healthcare sectors, including veterinary medicine. GPs classified as ‘resigned’ showed a reluctance to assume responsibility for AMR and Planetary Health in primary care, expressing frustration and pessimism.

Among GPs of the “resigned type,” the integration of existing knowledge and awareness about Planetary Health into consultations for justifying rational antibiotic prescribing was not consistently observed. These GPs had limited ideas on how to raise patient awareness for Planetary Health, citing time pressure and the fear of patronizing patients as barriers to engaging in more detailed discussions.

#### 3.4.3 Type 3: The unconscious and open-minded type

The “unconscious and open-minded type” comprised the largest group, with eight GPs possessing a medium level of prior knowledge about Planetary Health. Some were familiar with the term, and all were capable of establishing links between AMR and Planetary Health.

Within the “Type 3 GPs,” there was strong problem awareness. They believed that addressing the consequences of AMR at the planetary level was the responsibility of other stakeholders in the health care system and, in some cases, an individual duty.

Most of them reported practicing cautious antibiotic prescribing. These GPs reported bringing up Planetary Health and AMR during consultation when appropriate, citing time pressure, concerns about patronizing patients, and a lack of ideas on how to address the topic as limitations. Although not explicitly mentioning Planetary Health, some of these GPs discussed cautious antibiotic prescribing with patients, emphasizing the importance for the health of future generations.

General Practitioners of the “unconscious and open-minded type” considered themselves open to further engagement with Planetary Health and expressed interest in concrete advice on how to integrate it into GP consultations.

#### 3.4.4 Type 4: The motivated and resilient type

The “motivated and resilient GPs” demonstrated a deep understanding of the Planetary Health concept. Four GPs were assigned to this type.

They had a strong awareness of the extent of the AMR problem. These GPs primarily viewed themselves as responsible for their patients, while also recognizing the necessity of systemic changes to effectively address AMR and Planetary Health. Some GPs expressed pessimism about timely solutions for Planetary Health challenges and concerns about the difficulty of raising awareness among patients. However, despite these challenges, they exhibited resilience and motivation to actively promote Planetary Health.

These GPs expressed interest in further education and networking to enhance the collective ability to act. They reported regularly integrating Planetary Health and AMR topics into their consultation, with some describing the use of targeted co-benefit arguments when appropriate.

All “Type 4 GPs” emphasized the importance of conscious and rational prescribing. Additionally, some of them reported voluntary engagement in Planetary Health beyond their work context.

## 4 Discussion

To our knowledge, this is the first study to explore a GP perspective on AMR as a Planetary Health challenge. Previous research and publications have separately addressed AMR (1, 33–39) and Planetary Health (3, 40–44). Although there is an increasing body of evidence recognizing AMR as a Planetary Health concern (11, 12, 45–50), there is insufficient emphasis on primary care as a key setting for action.

### 4.1 Knowledge and problem awareness

Our study shows that the interviewed GPs were aware of AMR, and overall, approach the topic with a high level of seriousness. This aligns with the findings of the RAI study group, which observed GPs acknowledging the danger and recognizing the multifactorial genesis of emerging resistance (34). Most interviewed GPs were not familiar with the term “Planetary Health.'” This lack of familiarity is likely because the term is relatively new, and currently, there are limited opportunities for Planetary Health training for GPs in Germany (51–53).

Despite the increasing inclusion of Planetary Health in the curricula of medical students and other health care professionals (54), it is noteworthy that the majority of these courses are elective rather than an integral part of the core curriculum (55–59). Another potential factor contributing to the relatively low level of knowledge regarding the links between AMR and Planetary Health is the frequent absence of overarching Planetary Health terminology in research conducted in these fields (4, 60).

While a majority of publications on Planetary Health and AMR advocate for a unified approach to address both challenges simultaneously at a global scale (46, 48, 49, 61), others emphasize the need to tackle the intertwined issues at the national level, as seen in reports from Australia (45) or Germany (47). These publications primarily target policymakers or other decision-makers. Articles directly related to primary care providers regarding Planetary Health are rare and often address AMR without focusing on Planetary Health (1, 36, 37, 39) or concentrate on other Planetary Health-related topics, such as heat and other more direct health effects of the climate crisis (19, 41, 62).

Interestingly, most of the interviewed GPs could identify AMR as a Planetary Health challenge and readily establish the connections when prompted to do so. Since AMR is already being taken seriously, connecting the dots and framing AMR as a Planetary Health challenge could be a strategy to make Planetary Health more tangible for primary care providers and garner increased support from policymakers (16, 60). The “World Organization of National Colleges and Academic Associations of General Practitioners/Family Physicians” (WONCA) represents a good example of this approach. WONCA has recently adapted their “European Definition of General Practice/Family Medicine,” an important policy document for GPs all over Europe, by integrating the three topics One Health, Planetary Health and Sustainable Development Goals (SDGs) (63). Their new definition features these concepts as “bedrocks of family medicine” and therefore gives them a status of high importance. This can be influential for the alignment of future research and education programs, as it could put the focus on exploring the primary care perspective on Planetary Health.

Framing AMR as a Planetary Health challenge could also contribute to social tipping (15), by leveraging a shift in social norms among GPs toward rational antibiotic prescribing as a means to preserve Planetary Health (42).

### 4.2 Accountability

Many GPs attributed the responsibility for the development of AMR primarily to other medical specialties, such as veterinary medicine or inpatient care providers. However, this perception does not align with resistance data, as in Europe; overall, antibiotic consumption is higher in human medicine than in veterinary medicine (64). Additionally, the sectors cannot be viewed in isolation, as antibiotic use in animals and humans mutually reinforces the emergence of resistance in the respective organisms (65). Consequently, addressing AMR requires a collective and integrated approach.

Moreover, despite an overall decrease in antibiotic use in human medicine, there is a concerning rise in the use of broad-spectrum antibiotics, exacerbating the resistance situation (64). Another misconception is that AMR primarily emerges in hospital care. In Germany, 700–800 tons of antibiotics are used in human medicine annually, with 85% prescribed in outpatient care (66). In 2014, GPs and internal medicine specialists in primary care settings were responsible for 59% of antibiotic prescribing in outpatient care (66). This underscores the critical need for the primary care sector to recognize its significant role in shaping AMR development and to take ownership of corresponding responsibilities.

The majority of the interviewed GPs expressed a sense of accountability for addressing AMR with their patients or positioned themselves as generally open-minded to taking responsibility, including adopting more restrictive prescribing practices.

However, other studies have indicated that GPs are reluctant to change their prescription habits (36) and do not link the risk of AMR for individual patients to the risk for the community (67). This indicates an intention-behavior gap: a shift in intention, such as a desire to mitigate resistance development, does not automatically translate into a change in behavior, like altering prescription patterns (68). One possible explanation is that the most severe consequences of AMR and the climate crisis are often observed in settings beyond primary care, such as hospitals or countries with already high levels of antimicrobial resistance. Consequently, GPs may not receive direct positive reinforcement for prescribing antibiotics restrictively (69). According to Mentzel and Maun, the self-concept of autonomous entrepreneurship among GPs in Germany may also contribute to the intention-behavior gap (70). Entrepreneurial GPs face numerous challenges influencing their decision-making, including economic regulations and patient preferences. Consequently, they may make treatment decisions that contradict their fundamentally problem-aware stance on AMR (70).

Many GPs expressed worries and a sense of helplessness regarding the emergence of AMR and the future health effects of the climate crisis. According to Uzzell, when a problem is perceived as uncontrollable, inaction and the denial of personal involvement might reduce anxiety levels (69). This could explain why some interviewed GPs attributed responsibility to other stakeholders in the healthcare system. Another possible explanation for this attitude is the optimistic bias proposed by Uzzell, where individuals believe that negative events, such as concrete health threat from resistant germs, are more likely to affect others than themselves (69).

According to the political scientist Erica Chenoweth, when 3.5% of a population initiates social change, it can be sufficient to gain majorities for a particular purpose and transform the circumstances (71). GPs have the potential to serve as crucial change agents, playing a key role in “flipping the switch” and enhancing awareness of AMR as a Planetary Health challenge within the population they serve and among their peers.

Increasing GPs’ awareness of their potential to play a key role in Planetary Health is crucial. By providing holistic, long-term and patient-centered care, GPs can serve as a bridge between the health sector and the community and amidst the different medical specialties (43). It is important to consider the underestimation of general practice vulnerability to AMR, potential denial and refusal, and the intention-behavior gap when developing effective and sustainable strategies to empower GPs as resilient actors of change.

### 4.3 Addressing AMR and planetary health in practice

#### 4.3.1 Barriers in practice

In line with prior research (72), interviewees reported that they tend to prescribe antibiotics when patients expect them to do so. Research also suggests that doctors anticipate an improvement in the doctor-patient relationship when more medication is prescribed (67). Therefore, it is crucial to enhance GPs’ confidence in practicing restrained prescribing and provide guidance on effectively communicating the health benefits.

General Practitioners take into account patients’ socioeconomic backgrounds in their decision-making and arguments. Previous research indicated that patients with lower socioeconomic status are more likely to receive antibiotics (73). This tendency may be attributed to the fact that almost half of GPs do not practice participatory decision-making regarding antibiotic prescriptions, assuming their patients lack interest (34). This assumption could be rooted in a classist misconception that patients with lower socioeconomic backgrounds may not fully understand the complex Planetary Health implications of antibiotic prescribing (74, 75). To address this issue, it is essential to educate GPs on customizing their communication about Planetary Health topics for individual patients, taking into account their educational and social backgrounds, as well as personal biases.

Time constraints and economic pressures were mentioned as barriers to incorporating Planetary Health arguments and addressing AMR during patient consultations. This meets the findings of André et al. (76) and aligns with results from the RAI project, which indicate that a lack of time is a significant factor preventing discussions on AMR (34). Furthermore, doctors under distress and frustration are more likely to prescribe medication and to communicate less (77). Outpatient care operates within an economic framework characterized by capped budgets and per capita lump sums (78). The commercialization of healthcare, coupled with austerity measures, has compartmentalized the healthcare sector, impeding the integration of holistic approaches (79). Therefore, offering financial remuneration for counseling on AMR and other Planetary Health topics could serve as a beneficial transitional reform. However, it is acknowledged that a fundamental reorganization of the healthcare system is necessary, with a focus on population health outcomes such as Planetary Health mitigation measures, to ensure the well-being of both humans and the planet (80). This approach is exemplified by the AWMF guideline “Protection against the overuse and underuse of health care—deciding together” which explicitly considers the reduction of CO_2_ emissions as a key outcome (81).

#### 4.3.2 Suitable options for action

Aligned with the framework on “social accountable health care,” from the College of Family Physicians of Canada, GPs can address the link between AMR and Planetary Health at the micro, meso, and macro levels (82). The micro-level pertains to the GPs’ clinical environment or their practice (82). Our interviewees reported a cautious approach to prescribing antibiotics and self-reflection on AMR in their practice. In some cases, their knowledge, attitude, and practical skills were sufficient to address AMR as a Planetary Health challenges during consultations. Linking individual health benefits of specific behaviors to the reduction of AMR through the concept of co-benefits was identified as a promising strategy, aligning with recent publications on Planetary Health communication (42). Leveraging GPs’ high credibility and proximity to their patients (83) could create a social tipping effect, leading to increased awareness of AMR and Planetary Health among patients (15). Recognizing that changes in individual behavior alone may not be sufficient to address AMR, GPs could also be empowered to engage on the meso-level (82). Participants expressed interest in interprofessional exchange and training, which should be addressed through suitable social infrastructure and participatory educational offerings for GPs. Existing global networks, such as the Planetary Health Alliance (84), or regional organizations like the Planetary Health Academy (85), the German Climate Change and Health Alliance (86), or Health For Future groups in various European countries (87), serve as positive examples and could be expanded to reach both students and practicing physicians.

According to André et al. (76), GPs feel like there is a lack of clinical recommendations regarding the integration of Planetary Health into their practice. Therefore, the co-benefit-strategy can be employed to engage GPs who endorse EBM by demonstrating that addressing AMR in consultations or community exchanges can result in heightened acceptance or even a demand for more restrictive antibiotic prescriptions (88). The integration of Planetary Health into clinical guidelines could thus simultaneously raise awareness of the importance of the holistic view of health and make it feasible by linking it to concrete, evidence-based clinical advice. Additionally, the reduction of unnecessary prescriptions reduces the carbon footprint of general practice (89). Physicians can thus be motivated to practice EBM while contributing to Planetary Health, without additional efforts beyond their already overloaded daily routines.

Empowering GPs on a meso level can significantly amplify their efforts to raise awareness among patients and encourage action on the macro-level (43, 82). By leveraging GPs’ expertise in the interplay between AMR and Planetary Health, they can exert a positive influence on policy decisions that promote a healthier planet and enhanced human well-being. This engagement can occur at various levels, including local politics, medical associations, and professional colleges. An example is the German College of General Practitioners and Family Physicians (DEGAM), which is already actively involved in Planetary Health initiatives and welcomes further participation from GPs (90).

The four distinct types of GPs exhibit varying levels of knowledge, diverse approaches to addressing Planetary Health challenges ranging from frustration to resilience, and differing levels of practical skills and ideas. Applying the “Stages of Change” model, individuals can be positioned in different stages concerning a process of change, allowing for personalized and adapted interventions (91). Thus, the communication strategy and measures must be adapted to the target group (92). This concept can be applied to the various types of GPs, necessitating customized measures tailored to each type (refer to Table 1).

General Practitioners with a low level of knowledge about AMR and Planetary Health, such as those of the rejecting type, could benefit from easily accessible information materials. In a study by Kotcher et al. (93), health professionals expressed a desire for continuing professional education, policy statements, patient information materials, and trainings for effective patient communication regarding Planetary Health. To expand their knowledge, type 1 GPs could eventually profit from low threshold offers like podcasts, articles, or simple informative graphics in specialist magazines or the general press. Formats working with co-benefits-argumentation could strengthen their problem awareness and the feeling of accountability by illustrating the GPs’ advantage when maintaining Planetary Health. A survey conducted by the “Stiftung Gesundheit” (Health Foundation) on behalf of the Center for Planetary Health Policy suggested that a financial incentive system with changes to the charging system could be helpful (94). This approach could be expanded, e.g., to include education about AMR and Planetary Health.

According to Prochaska’s model of change theory, the stages of “consciousness raising” and “dramatic relief” are crucial for behavior change (91). In this context, “dramatic relief” refers to the process by which strong emotions about an issue, e.g., guilt or fear, are reduced with a feeling of relief when appropriate action can be taken (91). Applied to the different types of GPs, particularly those of type 2 and 3, “consciousness raising” could be achieved through formats aimed at fostering critical awareness for Planetary Health in primary care (95). Interventions that create emotional involvement, combined with tools offering actionable options, can lead to the so-called “dramatic relief,” thereby supporting change (91). This might involve empowering formats addressing GPs’ resignation, or interactive workshops, or roleplays triggering affect and anchor motivation. Simultaneously, GPs could benefit from trainings with a content focus on primary care and concrete options for action. This could include tips on working with co-benefits during consultations or promoting rational prescribing practice.

Additionally, both type 2 and type 3 GPs could benefit from training sessions, targeted quality circles and networking opportunities to consolidate their knowledge. Participating in a community with other GPs who share similar challenges and exchanging ideas with them could have an empowering effect. This community engagement is also proposed as a promising strategy in the context of climate communication, as it strengthens the willingness to cooperate (96). Activating GPs could also include empowering them to participate in designing training sessions regarding Planetary Health to raise their own ability to act. Initiatives like the “Planetary Health Academy” offer support and materials needed to create these workshops, closely adapted to the specific community’s needs (53).

Type 4 GPs could eventually be informed by updated specialist literature. Moreover, they have the opportunity to become educators through “Train the Trainer” courses (97). These courses may emphasize activating the community or peers by providing straightforward explanations of the interconnections between AMR and Planetary Health. Furthermore, type 4 GPs could benefit from confirmation, validation, and the visualization of their achievements to maintain their motivation and resilience (96). This could be achieved by using visual tools like apps or software programs, that enable monitoring progress and enhancements in addressing AMR or in incorporating adaptations that promote Planetary Health in one’s practice. Another approach could involve implementing feedback systems for trainers to highlight the significance of their advocacy work within the GP community, visualizing milestones in knowledge acquisition and practical skill development among participants.

Consideration should be given to the association between male gender, older age of GPs, and the location of practices in former East Germany, as these factors are linked to a higher likelihood of initiating antimicrobial therapy (98). This information should be taken into account when developing interventions.

### 4.4 Strengths and limitations

Our research was embedded within the broader RedAres study, providing a robust foundation with a diverse sample of participants across different regions and age groups. The theoretical framework and the mixed methods approach ensured a structural and comprehensive exploration of the research question. All participants belonged to the intervention group of the RedAres study. They engaged with the topics of rational antibiotic prescribing and AMR before and received individual feedback for their prescriptions. This exposure may have resulted in a higher level of knowledge and interest in AMR compared to other GPs. As participation in the interview was voluntary, we assume selection bias toward GPs who were more interested in Planetary Health and eventually had more prior knowledge. To mitigate this bias, financial incentives were offered. Additionally, a social desirability bias might have influenced participants to frame AMR as a major and urgent problem to align with the prevailing discourse.

Our study exclusively focused on GPs, excluding other medical professions in primary care. The digital format of the interviews could have posed a barrier for GPs without necessary technical equipment and might have affected the trust-building process between the interviewer and interviewee. While we employed maximal variation sampling for gender and region, we did not consider GPs’ age and work experience, factors that could impact attitudes and practical experiences of the interviewees.

## 5 Conclusion

General Practitioners generally demonstrated an awareness of AMR as a significant Planetary Health challenge. While some expressed a sense of accountability, others did not perceive primary care as being responsible. Many of the interviewed GPs conveyed a desire for additional education on preventing further AMR emergence while also safeguarding Planetary Health. Based on our findings, future research should foster the development and evaluation of tailored interventions and training programs to raise GPs awareness of the link between AMR and Planetary Health, and of the urgency to act. Encouraging EBM practices with a clear understanding of their impact on Planetary Health could be a straightforward individual-level measure. At the macro level, integrating Planetary Health outcomes into guidelines represents a significant step forward.

To empower GPs, fostering interprofessional exchange within local medical networks is essential. Interventions should leverage GPs’ potential as critical catalysts for change, enhancing their capacity to take meaningful action.

## Data availability statement

The raw data supporting the conclusions of this article will be made available by the authors, without undue reservation.

## Ethics statement

The studies involving humans were approved by Ethics Committee of the Medical Faculty, University of Wuerzburg. The studies were conducted in accordance with the local legislation and institutional requirements. The participants provided their written informed consent to participate in this study.

## Author contributions

PT: Writing – original draft, Writing – review & editing, Conceptualization, Data curation, Methodology, Resources, Visualization. AG: Resources, Writing – review & editing. IG: Resources, Writing – review & editing. JK: Resources, Writing – review & editing. AM: Resources, Writing – review & editing. GS: Writing – review & editing. E-MS-S: Writing – review & editing. CH: Supervision, Writing – review & editing. AS: Conceptualization, Data curation, Methodology, Resources, Supervision, Visualization, Writing – review & editing.
