# Supplementary material for: Addressing AMR and planetary health in primary care: the potential of general practitioners as change agents
Source: Front Public Health. 2024 Jul 31;12:1383423. doi: 10.3389/fpubh.2024.1383423 (PMC11322125; doi:10.3389/fpubh.2024.1383423)
Supplement: Supplementary file 1 [file Data_Sheet_1.docx]

Supplementary Material

Addressing AMR and Planetary Health in primary care: The potential of general practitioners as change agents

**Paula Tigges^1*^, Alexandra Greser^2^, Ildikó Gágyor^2^, Judith Kraft^1^, Andy Maun^4^, Guido Schmiemann^5^, Eva-Maria Schwienhorst-Stich^2,6^, Christoph Heintze^1^, Angela Schuster^1^**

^1^ Institute of General Practice, Charite University Hospital Berlin, Germany

^2^ Department of General Practice, University Hospital Wuerzburg, Wuerzburg, Germany

^3^ Department of General Practice, University Hospital Jena, Jena, Thuringia, Germany

^4^ Institute of General Practice / Primary Care, Faculty of Medicine and Medical Center, University of Freiburg, Germany

^5^ Department of Health Service Research, Institute for Public Health and Nursing Research, University of Bremen, Bremen Germany

^6^ Teaching Clinic of the Faculty of Medicine and Institute of Medical Teaching and Medical Education Research, University of Würzburg, Würzburg, Germany

***Correspondence:** [paula.tigges@charite.de](mailto:paula.tigges@charite.de)

# Supplementary Material

## Codebook
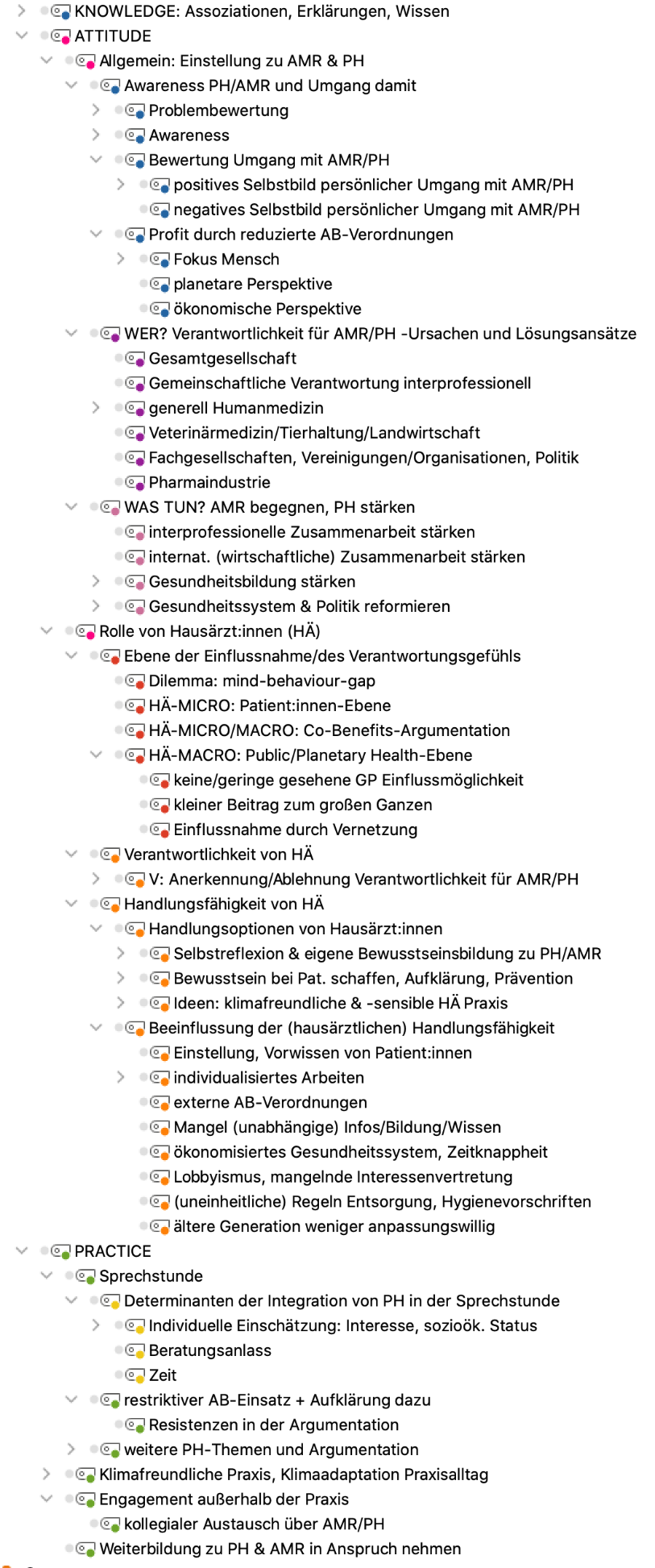


**Attachment 1.** Codebook with inductive and deductive categories
